# Massive Retinal Gliosis Mistaken as a Malignant Intraocular Tumor in Phthisis Bulbi

**DOI:** 10.3390/diagnostics12123090

**Published:** 2022-12-08

**Authors:** Soyoung Im, Hong Sik Park, Uiju Cho, Tae Yoon La

**Affiliations:** 1 Department of Hospital Pathology, St. Vincent’s Hospital, College of Medicine, The Catholic University of Korea, Seoul 16247, Republic of Korea; 2 Department of Ophthalmology and Visual Science, St. Vincent’s Hospital, College of Medicine, The Catholic University of Korea, Seoul 16247, Republic of Korea

**Keywords:** gliosis, massive retinal gliosis, intraocular tumor

## Abstract

Massive retinal gliosis (MRG) is a rare condition of non-neoplastic glial proliferation, which forms massive lesions that fill the eye. MRG is commonly associated with phthisis bulbi (a non-functional eye), congenital anomalies, or malformations. Herein, we report a case of massive retinal gliosis associated with a traumatic phthisis bulbi, which was initially mistaken as a malignant intraocular tumor and confirmed only after an eye enucleation. A 70-year-old woman presented with a protruding ocular mass in her left eye which had slowly grown for a year. She had phthisis bulbi in her left eye due to trauma during her childhood. An orbital CT revealed an intraocular mass lesion with calcifications, raising the possibility of retinoblastoma or other malignant intraocular tumors. Enucleation of the left eye globe was performed. Histopathologic examination revealed exuberant proliferation of the glial cells, metaplastic bone formation, hyalinized vessels, and hyperplasia of the retinal pigment epithelium, confirming the diagnosis of MRG. Although rare, the possibility of MRG should be considered as a differential diagnosis when encountering an intraocular mass lesion, as it can be misdiagnosed as a malignant tumor.

**Figure 1:**
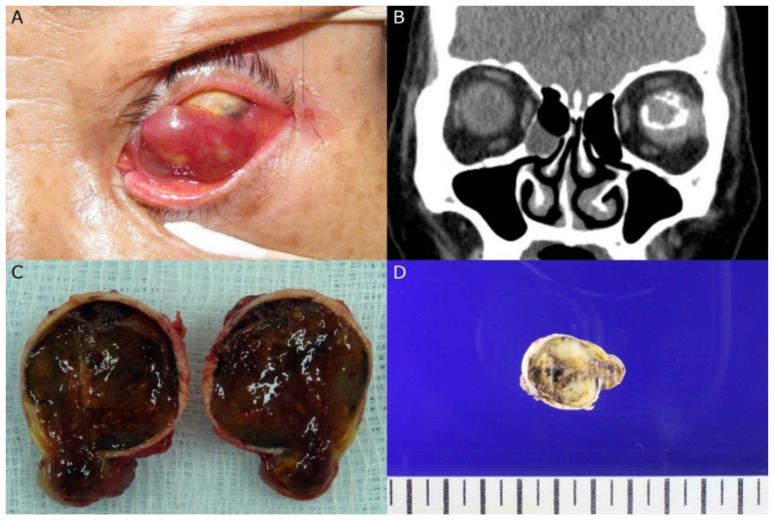
A 70-year-old female presented with a protruding mass lesion in her left eye. The mass had been growing slowly for several months. She had a history of phthisis bulbi due to trauma during childhood. (**A**) Ocular examination revealed a protruding mass lesion in the left eye with phthisis bulbi. (**B**) An intraocular mass filling the entire vitreous cavity was shown on orbital CT. Intratumoral calcification was noted. Initial impression was a malignant intraocular tumor such as retinoblastoma or uveal melanoma. (**C**) Enucleation of the eye was performed. A 2.7 × 2.3 cm sized mass with hemorrhage was filling the entire vitreous cavity and protruding into the anterior chamber. (**D**) On gross examination, a white and yellow colored solid mass with firm consistency was shown with focal hemorrhagic spots and a gritty cut surface.

**Figure 2:**
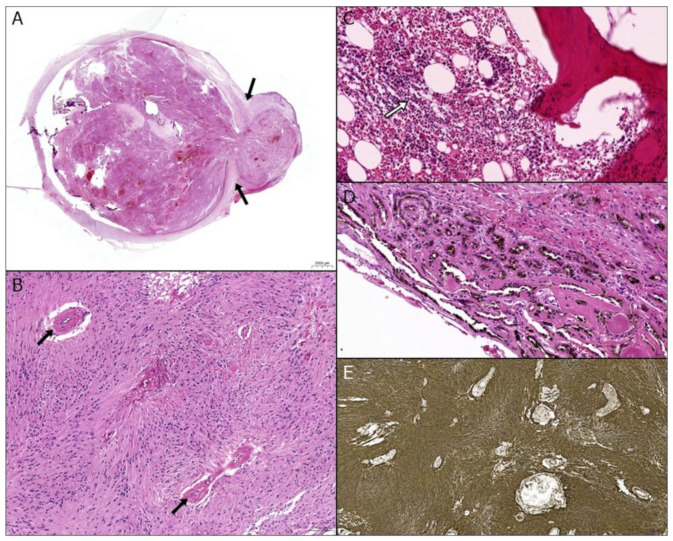
Microscopic findings. (**A**) Whole mount view of the left eye displayed a mass lesion filling the entire vitreous cavity and protruding into the anterior chamber (arrows) (H & E stain, ×44). (**B**) The mass lesion consisted of interweaving spindle cells forming fascicular and whirling patterns. Cytologic atypia was absent and mitotic activity was scanty (<1/50 HPF). Vessels with thick hyalinized walls (arrows) were scattered throughout the lesion. Note the predominance of spindle cells over vascular proliferation (H & E stain, ×100). (**C**) Metaplastic bone formation was present with intramedullary hematopoiesis (white arrow) (H & E stain, ×100). (**D**) Focal reactive hyperplasia of the retinal pigment epithelium was present at the base of the mass (H & E stain, ×200). (**E**) Diffuse intense positivity for GFAP immunohistochemical staining confirmed the exuberant proliferation of glial cells (×50). Pathologic diagnosis was massive retinal gliosis (MRG) with a Ki-67 labeling index of 10%. MRG is a rare benign intraocular lesion resulting from non-neoplastic glial cell proliferation [1]. Clinically, the mass forming feature of MRG can be easily mistaken for a malignant tumor, such as retinoblastoma, uveal melanoma, or intraocular metastatic tumor. The diagnosis can be made only after a histopathologic examination. The intense positivity of GFAP identified the proliferative spindle cells as glial cells, and eliminated the possibility of retinoblastoma or melanoma. Once the proliferating cells are confirmed as glial cells, the differential diagnosis can be narrowed down to retinal glial tumors and pseudotumors, such as retinal hemangioma, vasoproliferative tumors of the retina (VPTR), and MRG. In the previous literature, Housten et al. and Irvine et al. suggested that retinal hemangioma, VPTR, and MRG belonged to a spectrum of the same disease process; retinal hemangioma at one end of the spectrum with vascular-predominance, MRG at the other end with glial predominance, and VPRT between the two ends with both vascular and glial components represented [2,3]. In this case, hyalinized blood vessels were scattered throughout the lesion. However, the exuberant glial proliferation was much more predominant. Three histopathologic criteria for MRG diagnosis have been described by Yanoff et al.: focal or total retinal replacement by the proliferating glial tissue, atypical blood vessels within the tumor, and obliteration of the normal retinal architecture by the proliferating glial tissue [1]. All features were clearly displayed in the present case. MRG is considered as a longstanding tissue response process following retinal injury, such as chronic inflammation, trauma, glaucoma, vascular disorders, and congenital anomalies [1,2,4,5]. The clinical setting of the present case with phthisis bulbi due to trauma was also compatible with MRG. Although MRG is a rare non-neoplastic lesion, it should always be considered as a differential diagnosis when encountering an intraocular mass. Being aware of the possibility can facilitate making the correct diagnosis, as it can be mistaken for a malignant tumor.
